# Stapling of the botulinum type A protease to growth factors and neuropeptides allows selective targeting of neuroendocrine cells

**DOI:** 10.1111/jnc.12284

**Published:** 2013-05-20

**Authors:** Jason Arsenault, Enrico Ferrari, Dhevahi Niranjan, Sabine A G Cuijpers, Chunjing Gu, Yvonne Vallis, John O'Brien, Bazbek Davletov

**Affiliations:** * MRC Laboratory of Molecular Biology, NeurobiologyCambridge, UK; † School of Life sciences, University of LincolnLincoln, UK; ‡ Department of Biomedical Sciences, University of SheffieldSheffield, UK

**Keywords:** botulinum, growth factor, neuroendocrine tumor, neuropeptide, SNAP25, targeting

## Abstract

Precise cellular targeting of macromolecular cargos has important biotechnological and medical implications. Using a recently established ‘protein stapling’ method, we linked the proteolytic domain of botulinum neurotoxin type A (BoNT/A) to a selection of ligands to target neuroendocrine tumor cells. The botulinum proteolytic domain was chosen because of its well-known potency to block the release of neurotransmitters and hormones. Among nine tested stapled ligands, the epidermal growth factor was able to deliver the botulinum enzyme into pheochromocytoma PC12 and insulinoma Min6 cells; ciliary neurotrophic factor was effective on neuroblastoma SH-SY5Y and Neuro2A cells, whereas corticotropin-releasing hormone was active on pituitary AtT-20 cells and the two neuroblastoma cell lines. In neuronal cultures, the epidermal growth factor- and ciliary neurotrophic factor-directed botulinum enzyme targeted distinct subsets of neurons whereas the whole native neurotoxin targeted the cortical neurons indiscriminately. At nanomolar concentrations, the retargeted botulinum molecules were able to inhibit stimulated release of hormones from tested cell lines suggesting their application for treatments of neuroendocrine disorders.

Neuroendocrine tumors (NETs) arise from cells of the endocrine and nervous systems. NETs are present not only in endocrine glands that secrete hormones but can also be localized throughout the body, for example, in the gastrointestinal tract, pancreatic islets, adrenomedulla, thymus, pituitary and lung tissues (Langley 1994). NETs can also arise from Schwann cells and neurons, forming schwannomas and neuroblastomas. Despite different embryological lineages, NETs have similar phenotypic characteristics and often exhibit excessive secretion of various neurotransmitters and hormones (Bajohrs *et al*. 2005; Ferolla *et al*. 2008). It is well known that excessive release of catecholamine, acetylcholine, insulin, and corticotropin from NETs can wreak havoc on endocrine homeostasis and thus new approaches to control secretory activity of neuroendocrine cells are urgently required (Batcher *et al*. 2011; Prejbisz *et al*. 2011; Kirmani 2012; Kroesen *et al*. 2012; Low *et al*. 2012; Tritos and Biller 2012). Here, we used a botulinum-based strategy to investigate targeting of a selection of widely used model neuroendocrine cells: mouse neuroblastoma (Neuro2a), insulinoma (Min6), pituitary adenoma corticotrophs (AtT-20), rat pheochromocytoma (PC12), and human neuroblastoma (SH-SY5Y) cells (Schiavo *et al*. 2000; Foster *et al*. 2006; Foster and Chaddock 2010; Pickett and Perrow 2011; Davletov *et al*. 2012).

Secretory activity of neuroendocrine cells and neurons relies on soluble *N*-ethylmaleimide sensitive fusion protein-attachment protein receptors (SNAREs): syntaxin 1, synaptosomal-associated protein 25 (SNAP25), and synaptobrevin (Fasshauer *et al*. 1998; Sutton *et al*. 1998; Südhof and Rothman 2009; Gao *et al*. 2012). These three proteins form a tight complex that allows fusion of secretory vesicles with the plasma membrane in both neurons and endocrine cells. Botulinum neurotoxin type A (BoNT/A), produced by a Clostridium bacterium carries a protease that selectively cleaves SNAP25 thereby blocking neuronal secretion for several months (Meunier *et al*. 2003; Davletov *et al*. 2005). The native BoNT/A recognizes specifically neurons via its receptor-binding domain and can then deliver its SNAP25 protease into the intra-neuronal environment (Yowler *et al*. 2002; Mahrhold *et al*. 2006; Binz and Rummel 2009). As neuroendocrine cells lack neuron-specific gangliosides, NET cells are normally resistant to the external application of BoNT/A (Yowler *et al*. 2002). However, in experimental settings, expression or delivery of the botulinum protease into NET cells leads to SNAP25 cleavage resulting in the blockade of hormonal secretion (Chaddock *et al*. 2000a,b; Bajohrs *et al*. 2004; Foster *et al*. 2006). Here, we address the possibility to deliver the botulinum protease to NET cells using a selection of growth factors and neuropeptides by exploiting a novel protein stapling method.

The most straightforward strategy for targeting a bacterial enzyme into desired cells would be to directly fuse it to a chosen ligand, provided the ligand is amenable for bacterial expression and the new structure permits receptor binding. In the case of the botulinum protease, its large size, its dependence on additional structural units for translocation into the neuronal cytosol, and its variable production yields forced us to explore a new approach. We recently introduced a so-called ‘protein stapling’ technology where a complex protein can be produced independently and then ‘stapled’ to a targeting unit (Darios *et al*. 2010; Ferrari *et al*. 2012). We demonstrated that the BoNT/A1 Botulinum enzymatic and Translocation domains (BoT), linked by a disulfide bond, could be functionally produced in *E.coli* as required for stapling (Darios *et al*. 2010). Here, we prepared a range of growth factors: epidermal growth factor (EGF), tumor necrosis factor α (TNFα), ciliary neurotrophic factor (CNTF); and neuropeptides: dermorphine (Dermo), dynorphin 17 (Dyn17), corticotropin-releasing hormone (CRH), substance P (SP), somatostatin, for parallel stapling to our standardized botulinum construct.

Our side-by-side tests on a selection of NETs demonstrated that growth factors and neuropeptides could target distinct tumor cells with the internalization of the botulinum enzyme evidenced by proteolysis of the SNAP25 protein responsible for exocytosis of neurotransmitters and hormones. The observed cell selectivity was further confirmed using BoT directly fused with the EGF and CNTF ligands, which could be produced in *E. coli* as fusion proteins. Our results show that the stapling technology allows not only parallel production of functional biological molecules but also their greater diversity in exploration of cell-targeting strategies.

## Materials and methods

### Protein production and stapling reactions

All proteins were expressed in the BL21 strain of *E. coli* as glutathione S-transferase C-terminal fusions cleavable by thrombin. The Botulinum light chain and Translocation domain (BoT) of the botulinum type A1 strain fused to SNAP25 (Staple), and the syntaxin peptide (stapling peptide) were prepared as previously described (Darios *et al*. 2010; Ferrari *et al*. 2012). The growth factors and neuropeptides’ DNA were synthesized by Eurogentec (Belgium) and cloned into pGEX-KG vectors. The plasmid for expression of synaptobrevin-fused ligands were generated as follows: rat synaptobrevin 2 (amino acids 25–84) was inserted into the pGEX-KG vector between *Bam*HI and *Eco*RI sites and the DNA sequences for human CRH (1–41), EGF (1–53), CNTF (1–200), dynorphin (1–17), and TNFα (57–233), each carrying a preceding double repeat GGSGG spacer sequence, were inserted into the XhoI site at the 3′ end of the synaptobrevin sequence. Rat syntaxin 3 (195–253) was used to construct the syntaxin-linked CRH in the same manner. For direct fusion to the BoT protein, CNTF, CRH, EGF, TNFα, and SP (1–11) were inserted between two XhoI sites in the pGEX-KG vector carrying the BoNT/A (1–872) sequence (Darios *et al*. 2010). All inserted DNAs were verified by sequencing using pGEX-3 and pGEX-5 primers (Source Bioscience, Nottingham, UK). Proteins were expressed and purified as previously described (Darios *et al*. 2010). Protein concentrations were determined using BCA Protein Assay Kit (Thermo scientific, Loughborough, UK). Syntaxin-SP (Ac-EIIKLENSIRELHDMFMDMAMLVESQGEMIDRIEYNVEHAVDYVE-Ahx-Ahx-RPKPQQFFGLM-NH_2_), synaptobrevin-SP (Ac-RLQQTQAQVDEVVDIMRVNVDKVLERDQKLSELDDRADALQAGAS-Ahx-Ahx-RPKPQQFFGLM-NH_2_), synaptobrevin-somatostatin (sequence: Ac-RLQQTQAQVDEVVDIMRVNVDKVLERDQKLSELDDRADAL-Ahx-Ahx-AGCKNFFWKTFTSC-OH), dermo-synaptobrevin (Y(D-Ala)FGYPS-Ahx-Ahx-RLQQTQAQVDEVVDIMRVNVDRVLERDQRLSELDDRADALQAGAS-NH_2_) were prepared by Peptide Synthetics UK, dissolved in dimethyl sulfoxide, and diluted in Buffer A (100 mM NaCl, 20 mM HEPES, pH 7.4) to 1 mg/mL. Assembly reactions were performed in Buffer A supplemented with 0.4% n-octyl-β-d-glucopyranoside (Calbiochem, San Diego, CA, USA) with ratios of BoT-Staple/stapling peptide/ligand at 5 : 7 : 7. Protein assembly reactions were at 20°C for 1 h. Sodium dodecyl sulfate-resistant, and irreversibly assembled protein complexes were confirmed on sodium dodecyl sulfate–polyacrylamide gel electrophoresis (SDS–PAGE) gels migrated at 4°C. Proteins were added to cell cultures and incubated for 42 h. In competition assays an excess of ligand was incubated 15 min prior to the administration of the botulinum compounds. Human EGF was from Roche (Burgess Hill, UK) and human CNTF was from Invitrogen, Paisley, UK.

### Cell culture

Cells were grown in a 37°C incubator at 5% CO_2_. Neuro2A and SH-SY5Y cells were grown in low glucose Dulbecco's Modified Eagle Medium (DMEM, Gibco, Rockville, MD, USA) supplemented with 10% fetal calf serum (FCS Fetalclone1, HyClone) and 1% Penicillin/Streptomycin (P/S, Invitrogen). Every 3–4 days, cells were washed with Phosphate Buffer Solution (PBS) and resuspended in culture medium using flow pressure, then counted using a hemacytometer. Cells were plated in at 1 × 10^6^ cells per 9 cm culture dish (BD Falcon) or at 1 × 10^5^ cells per well in uncoated 24-well plates (BD Falcon). PC12 cells were grown in low glucose DMEM supplemented with 10% equine serum (ES, HyClone), 5% FCS and 1% P/S in pre-coated type IV collagen coated 75 cm^2^ flasks (BD bioscience, San Jose, CA, USA). Cells passage was passed once per week and the medium changed at midpoint between passages. During passage, cells were washed in PBS, then treated with trypsin, and plated at 3 × 10^6^ cells per flask or at 2 × 10^5^ cells per well on poly-l-lysine (Invitrogen) and collagen (Invitrogen) 25 : 1 coated 24-well plates. Min6 cells were grown in high glucose DMEM supplemented with 15% FCS, 1% P/S and 250 nM β-mercaptoethanol (VWR International, Lutterworth, UK). Cells were passed every 3–4 days, trypsin-treated and plated at 1.5 × 10^6^ cells per 9 cm culture dish or 1.5 × 10^5^ cells per uncoated 24-well plates. AtT-20 cells were grown in F12K medium supplemented with 15% ES, 2.5% FCS and 1% P/S. Cells were passed every 3–4 days and grown in suspension in uncoated 75 cm^2^ culture flasks (BD Falcon, San Jose, CA, USA) or plated at 2 × 10^5^ cells per well on poly-l-lysine/collagen 25 : 1 coated 24-well plates. Sprague–Dawley rat primary cortical neurons were prepared as previously reported (Darios *et al*. 2010). Briefly, embryonic day 16 brain cortices were dissected and the tissue incubated in papain (Worthington, Lakewood, NJ, USA) for 20 min at 37°C. Following dissociation, the suspension was filtered, centrifuged and precipitated cells plated at the density of 1 × 10^5^ cells/well in 24-well plates coated with poly-d-lysine 50 μg/mL (Sigma-Aldrich, Dorset, UK) and laminin 20 μg/mL from Engelbreth-Holm-Swarm murine sarcoma basement membrane (Sigma-Aldrich), with or without glass coverslips. Cells were maintained in Neurobasal medium (Gibco) supplemented with 1% B27 (Gibco), 1% P/S, and 400 μM l-glutamine (Gibco). Half of the medium was changed every 3–4 days and cultures were tested at least 1 week after plating and kept for a maximum of 4 weeks.

### Immunostaining and confocal microscopy

Cells grown on coated coverslips in 24-well plates were treated with or without BoT molecules 42 h before being fixed in 4% paraformaldehyde in PBS for 20 min at 20°C. The cells were further washed three times with PBS for 5 min and incubated 10 min in 10 mM NH_4_Cl. The cells were then incubated for 30 min in a permeabilization solution composed of 0.1% Triton X-100, 5% bovine serum albumin (BSA, Sigma-Aldrich) in PBS. Permeabilization solution was then replaced with 5% BSA in PBS containing appropriate primary antibodies. Mouse monoclonal anti-SNAP25 (SMI81, Novagen, Feltham, UK), rabbit polyclonal anti-cleaved SNAP25 antibody (Ekong *et al*. 1997; Antonucci *et al*. 2008; Ferrari *et al*. 2011), and mouse monoclonal anti-MAP2 (clone AP20, Millipore, Feltham, UK) were used at 1 : 500 dilutions. The wells were washed three times in PBS, then incubated for 30 min with Alexa Fluor® 488 goat anti-mouse IgG and/or Alexa Fluor® 594 goat anti-rabbit IgG diluted 1 : 800 in 5% BSA in PBS. Wells were washed three times with PBS. Coverslips were overturned onto Vectashield mounting medium, sealed, and visualized on Zeiss 710 confocal microscope at 63× and 20× magnification. The fluorescent gain intensities and pinhole size (1AU) were identical between experimental samples. Colocalization was quantified using the Zeiss software (Carl Zeiss Ltd., Cambridge, UK). Pictures for quantification were taken from random fields in three different regions of each glass coverslips.

### Western immunoblotting

Following treatment of cells with BoT molecules for 42 h, the wells were washed once with PBS before addition of 100 μL of loading buffer (56 mM sodium dodecyl sulfate, 0.05 M Tris-HCl, pH 6.8, 1.6 mM UltraPure EDTA (Gibco, Paisley, UK), 6.25% glycerol, 0.0001% bromophenol blue). One unit of benzonase (Novagen) supplemented with 1 μL of 1 M MgCl_2_, was added to each well and plates were shaken at 1500 rpm for 10 min. Samples were boiled for 1 min at 95°C and then run on 12% Novex SDS–PAGE gels (Invitrogen). Running time was doubled for SMI81 blotting to better visualize both cleaved and intact SNAP25. Following separation, proteins were transferred onto Immobilin-P polyvinylidene difluoride membranes, and then incubated for 30 min in blotting solution (5% milk, 0.1% TWEEN 20 in PBS). The above-mentioned primary antibodies and rabbit polyclonal anti-syntaxin 1b (Synaptic Systems, Goettingen, Germany) were added at 1 : 3000 dilutions to the blotting solution and incubated for 1 h. Membranes were washed three times in 0.1% TWEEN 20 in PBS for 5 min and then incubated for 30 min in the blotting solution containing secondary peroxidase-conjugated goat anti-mouse or anti-rabbit antibodies (Thermo Scientific), respectively. Membranes were washed three times for 5 min in 0.1% TWEEN 20 in PBS. Immunoreactive protein bands were visualized using SuperSignal West Dura Extended Duration solution (Thermo Scientific, Cramlington, UK) with exposure to Fuji Medical X-Ray films (Fuji, Ross-on-wye, UK). Band intensities were quantified as previously described (Arsenault *et al*. 2010).

### Release assays

^3^H-norepinephrin release assay was performed as described elsewhere (Taupenot 2007). Briefly, 2 × 10^5^ PC12 cells were grown on poly-l-lysine and collagen-coated 24-well plates and incubated with BoT molecules for 42 h. Cells were incubated with ^3^H-norepinephrin (Perkin Elmer, Waltham, MA, USA) for 90 min at 37°C in the complete medium, washed with pre-warmed basal buffer (150 mM NaCl, 5 mM KCl, 2 mM CaCl_2_, 10 mM HEPES, pH 7.4) and then stimulation was triggered using potassium chloride buffer (100 mM NaCl, 55 mM KCl, 2 mM CaCl_2_, 10 mM HEPES, pH 7.4) for 15 min. Medium was removed for scintillation radioisotope counting. Remaining cells were resuspended in the basal buffer supplemented with 0.2% Triton X-100. All samples were mixed with scintillation fluid (4 : 1 ratio) and counted with Perkin Elmer Tri-Carb 2910TR liquid scintillation counter. Percentage of release was equal to soluble fraction (released) divided by the sum of the soluble fraction and the insoluble fraction (unreleased). ACTH release was performed using an enzymatic immunoassay kit (EIA, Pheonix Pharmaceuticals, Karlsruhe, Germany). Briefly, AtT-20 cells were treated with the botulinum molecules for 42 h before assay were washed twice with the basal buffer, and then incubated for 10 min at 37°C either in a pre-warmed incomplete F12K medium (basal condition) or pre-warmed incomplete F12K medium supplemented with 10 μM bovine CRH (Sigma-Aldrich; stimulated condition). Media were removed, briefly centrifuged, and supernatants were added to 96-well immunoassay plates pre-coated with secondary antibody. The released adrenocorticotropic hormone (ACTH) peptide competes for a biotinylated ATCH peptide with the primary antibody and signal is revealed by a decrease in bound streptavidin-horseradish peroxidase. All data points were performed in triplicate.

### Statistical analysis

All experiments were performed at least in triplicate. Results are presented as mean ± standard deviation (SD). Data analysis was performed using Graphpad Prism 5.0 (La Jolla, CA, USA). The unpaired 2-tailed Student's *t*-test was used for comparison. A *p* value of < 0.05 was considered statistically significant.

## Results

### Retargeting the botulinum protease

**Fig. 1:**
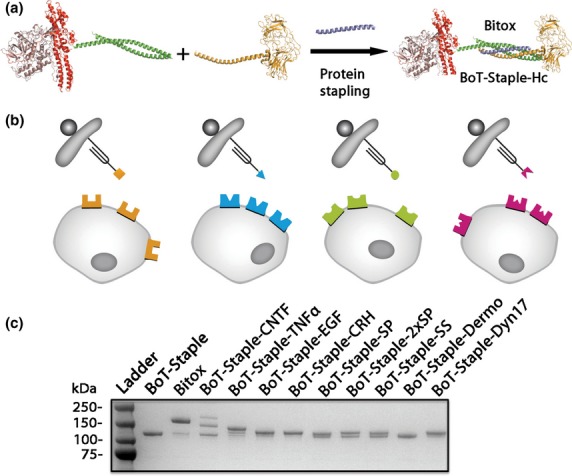
The protein stapling technique used for cell targeting. (a) Schematic representation of protein stapling using the Botulinum enzymatic and translocation domains (BoT) (the protease domain, brown; the translocation domain, red, and a SNAP25 linker, green), the native receptor-binding domain (Hc) (yellow, contains a synaptobrevin linker) and the stapling peptide (syntaxin-derived, blue). Addition of the stapling peptide results in formation of an irreversible botulinum construct (Bitox). (b) A diagram showing the cell targeting approach utilizing a range of ligands. (c) Coomassie-stained sodium dodecyl sulfate–polyacrylamide gel electrophoresis gel showing an array of BoT-Staple-constructs carrying the indicated targeting domains: ciliary neurotrophic factor (CNTF); tumor necrosis factor α (TNF-α); epidermal growth factor (EGF), corticotropin releasing hormone (CRH), substance P (SP), double substance P (2 × SP); somatostatin (SS); dermorphin (Dermo); dynorphin 17 (Dyn17). BoT-Staple is a control construct without a targeting moiety.

A schematic representation of the protein stapling technique is outlined Fig. 1a, where the BoT (aa 1-872) portion of the BoNT/A1 is stapled to the native receptor-binding domain of BoNT/A1, resulting in a functional neuronal blocking construct (Darios *et al*. 2010). In a similar fashion, new targeting domains were attached to the BoT unit to target the cognate cell surface receptors (Fig. 1b). The assembly of diverse targeting domains with the BoT unit was visualized in a Coomassie-stained SDS–PAGE gel (Fig. 1c).

### Cleavage of cellular SNAP25 by targeted botulinum constructs

**Fig. 2:**
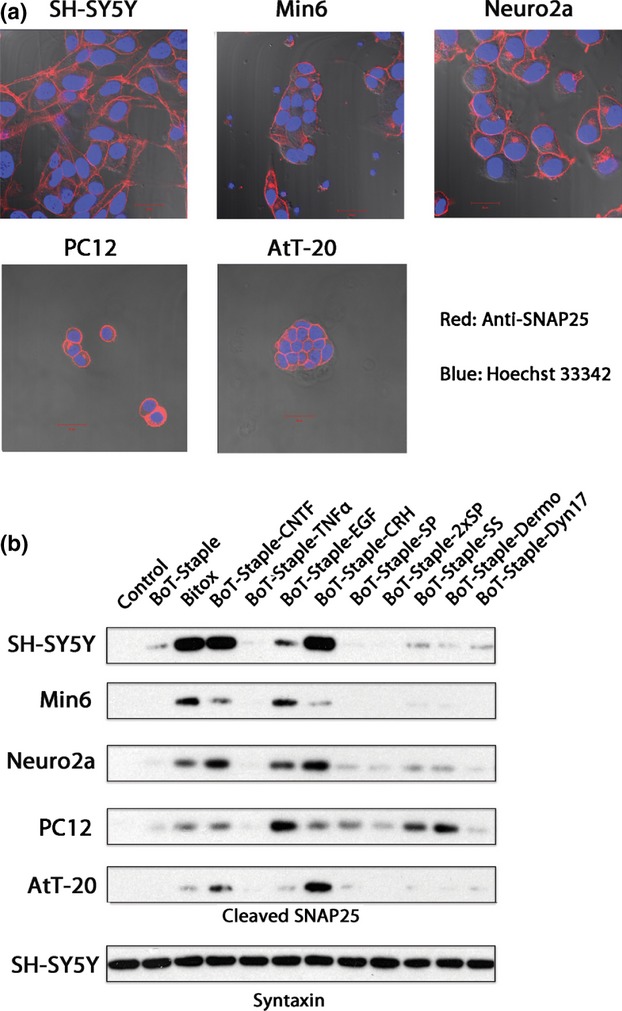
Targeting of neuroendocrine tumor cells using stapled botulinum constructs. (a) SNAP25 expression visualized by immunocytochemistry in the indicated cells: human neuroblastoma SH-SY5Y, mouse insulinoma Min6, mouse neuroblastoma Neuro2a, rat pheochromocytoma PC12, and mouse pituitary adenoma corticotroph AtT-20 cell. (b) Western blotting of the cleaved SNAP25 product in tested cells reveals selective botulinum activity for newly designed constructs. Untargeted Botulinum enzymatic and translocation domains (BoT)-Staple was used as a control, followed by Bitox and ligand-targeted BoTs as indicated in Fig. 1c. Total syntaxin immunoreactivity, shown here for SH-SY5Y cells, was routinely used as a loading control.

The SNAP25 protein, responsible for hormone secretion and located on the inner leaflet of the plasma membrane, can be visualized in five tested NET cells (pheochromocytoma PC12, insulinoma Min6, neuroblastomas SH-SY5Y and Neuro2A, and pituitary AtT-20) (Fig. 2a). The targeted BoT versions were incubated with the cells for 42 h and the intracellular cleavage of SNAP25 was examined using a polyclonal antibody that recognizes only its cleaved end, specifically the TRIDEANQ amino acid sequence (Fig. 2b) (Ekong *et al*. 1997). Because we have used the same starting concentration of the BoT unit, the difference in SNAP25 cleavage was exclusively because of the efficiency of the ligands to target cells, and delivered the botulinum protease into the cytosol of NET cells. The latter step involves internalization of the cognate receptors, a pH-dependent translocation of the botulinum protease, and the protease activity itself (Schiavo *et al*. 1993; Binz and Rummel 2009).

We observed that Bitox, which carries the native BoNT/A targeting domain, could cleave SNAP25 in SH-SY5Y neuroblastoma cells, which is in accord with the ability of native BoNT/A to target these cells (Darios *et al*. 2010; Thirunavukkarasusx *et al*. 2011). BoT constructs stapled to CNTF (aa 1-200) and CRH (aa 1-41) were able to cleave SNAP25 in SH-SY5Y cells as efficiently as Bitox while EGF exhibited a lower targeting capacity (Fig. 2b). In the Min6 insulinoma cells the pattern changed, the BoT-Staple-EGF was as efficient as Bitox whereas CNTF and CRH moieties were less effective. It has been reported that insulinoma cells are refractory to BoNT/A binding (Boyd *et al*. 1995); however, our highly sensitive cleaved-SNAP25 immunoassay does reveal some efficacy of botulinum-based targeting (Bitox) (Fig. 2b). In the Neuro2A cells, CNTF and CRH targeted BoT better than the native botulinum targeting part (Bitox) in agreement with a reduced ability of native BoNT/A to bind these cells (Yowler *et al*. 2002). In the PC12 pheochromocytoma cells, BoT-Staple-EGF was most efficient with somatostatin and dermorphin exhibiting next in rank efficiency. Interestingly, C-terminally amidated substance P and dermorphin demonstrated a significant activity, as well as CRH-linked BoT, suggesting the presence of cognate receptors in these cancer cells. Finally, in the AtT-20 adenoma cells the CRH-directed complex was far superior to other ligands as would be expected from pituitary corticotroph cells (Fig. 2b).

### Fully recombinant EGF and CNTF botulinum chimeras validate the observed cell specificity of the targeting domains

**Fig. 3:**
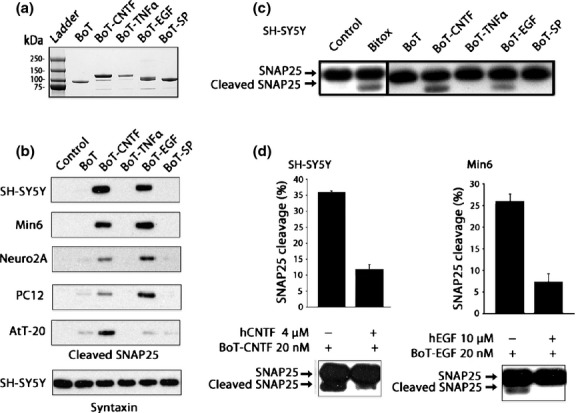
Targeting of neuroendocrine tumor cells using recombinant botulinum constructs. (a) Coomassie-stained sodium dodecyl sulfate–polyacrylamide gel electrophoresis gel showing recombinant proteins consisting of Botulinum enzymatic and translocation domains (BoT) fused directly to the indicated targeting domains: ciliary neurotrophic factor (CNTF); tumor necrosis factor α (TNF-α); epidermal growth factor (EGF), and substance P (SP). BoT is a recombinant protein carrying the botulinum protease and the translocation domain without a targeting moiety. (b) Immunoblot showing the botulinum activity of recombinant botulinum molecules (10 nM) as revealed using the cleaved SNAP25 antibody. Total syntaxin immunoreactivity was used as a loading control. (c) Monoclonal anti-SNAP25 antibody (clone SMI81) was used to confirm specificity of botulinum retargeting using CNTF and EGF to SH-SY5Y cells. (d) Bar charts showing that application of CNTF on SH-SY5Y or EGF on Min6 cells lead to a decrease in cleaved SNAP25 caused by BoT-CNTF and BoT-EGF, respectively (± SD). Representative immunoblots are shown below the bar charts.

Next, we recombinantly fused CNTF, EGF, CRH, TNFα, dynorphin 17, and substance P to the C-terminal end of the BoT unit. We did not prepare fusion of dermorphin as it requires free N-terminus for binding to opioid receptors (Amiche *et al*. 1988). Fusion of ligands to the original BoT unit led a 3- to 10-fold drop in protein yields (0.5–1.5 mg per liter of bacterial culture), while BoT-CRH and BoT-dynorphin 17 were not produced at detectable levels. Fig. 3a shows migration pattern of the recombinantly fused chimeras on a Coomassie-stained SDS–PAGE gel. Analysis of SNAP25 cleavage following their application (10 nM) for 42 h on the NET cells demonstrated similar pattern of activity compared to the stapled BoT molecules (Figs. 3b and 2b). In human SH-SY5Y cells, BoT-CNTF has a superior efficacy closely followed by BoT-EGF. Min6 cells display a reverse trend with BoT-EGF being superior. Neuro2A, PC12 and AtT-20 cells also exhibited SNAP25 cleavage in a specific way when treated with the EGF- and CNTF-BoT fusions. Overall, we conclude that when direct recombinant fusions can be obtained, the targeted BoT molecules show similar targeting pattern to that obtained with the stapled products. We did not observe a SNAP25 cleavage with the BoT-substance P fusion protein, as would be expected because of the lack of C-terminal amidation (Fisher *et al*. 1996), demonstrating that post-translational modifications need to be taken into account when re-targeting the botulinum protease toward desired cells.

To ascertain targeting specificity we used a monoclonal anti-SNAP25 antibody (clone SMI81) that recognizes both native and cleaved products. Among the recombinant chimeras, CNTF and EGF again were the most active ligands to direct the botulinum protease into the SH-SY5Y cells (Fig. 3c). The efficacy of these two growth factors was also confirmed with the SMI81 monoclonal antibody when rat cortical neuronal cultures were treated with the full range of stapled botulinum constructs (Figure S1). Next, we performed competition experiments using the native ligands. Fig. 3d (left panel) shows that the application of 4 μM human CNTF 15 min before the addition of 20 nM BoT-CNTF to SH-SY5Y cells resulted in a reduced cleavage of SNAP25. Similarly, application of 10 μM human EGF 15 min before the addition of 20 nM BoT-EGF to Min6 cells led to reduction in SNAP25 cleavage (Fig. 3d, right panel). The two targeted BoTs thus compete for binding sites with the native growth factors, indicating that ligand interactions with cognate EGF and CNTF receptors are necessary for the observed botulinum activity.

### Botulinum–based inhibition of hormone release

**Fig. 4:**
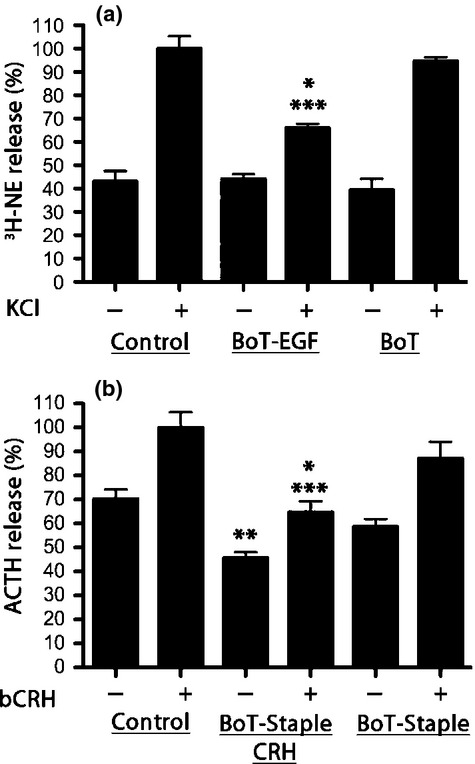
Inhibition of exocytosis using retargeted botulinum molecules. (a) A significant reduction in KCl-stimulated ^3^H-norepinephrine release was observed when PC12 cells were pre-treated with Botulinum enzymatic and translocation domains (BoT)-epidermal growth factor compared to untreated cells (*p < 0.01) or untargeted BoT (***p < 0.005). (b) A significant reduction in ACTH release was observed when AtT-20 cells were treated with BoT-Staple- corticotropin releasing hormone (CRH) compared to untreated cells both in the case of basal (**p < 0.02) and stimulated secretion (bovine CRH, bCRH; ***p < 0.005). BoT-Staple-CRH caused a significant reduction in ACTH release when compared to the untargeted BoT-Staple in the case of basal (**p < 0.02) and bCRH-stimulated secretion (*p < 0.05).

Because SNAP25 is obligatory for calcium-dependent exocytosis, the observed cleavage by the botulinum protease should result in inhibition of secretion of hormones and neurotransmitters. Pheochromocytoma cells serve as a model of regulated secretion and represent a valid medical target as a result of their excessive release of catecholamines (Martin and Grishanin 2003). We incubated PC12 cells in the presence of 20 nM BoT-EGF, or untargeted BoT, for 42 h and then measured the uptake and release of ^3^H-catecholamine. The BoT molecules did not affect total ^3^H-catecholamine uptake (Figure S2), whereas BoT-EGF caused a 34% reduction in K^+^-induced catecholamine secretion compared to control (Fig. 4a, *p* < 0.01). In contrast, Bitox at 20 nM concentration did not attenuate the release of catecholamine from PC12 cells (data not shown). In a previous study, very high doses of BoNT/A were required to lower catecholamine release which can be explained by the lack of high affinity binding sites on PC12 cells for the native botulinum molecule (Shone and Melling 1992). We also evaluated the action of CRH-targeted botulinum protease on secretion of ACTH from pituitary AtT-20 cells, a model for Cushing's disease (Bangaru *et al*. 2010). Cells were pre-treated with stapled BoT-Staple-CRH or an untargeted control (BoT-Staple) at 10 nM for 42 h. Fig. 4b shows that the AtT-20 cells exhibited a high rate of basal secretion of ACTH that was significantly reduced following application of BoT-Staple-CRH as measured by enzymatic immunoassay (*p* < 0.005). Furthermore, ACTH release triggered by native CRH was reduced by 36% following treatment with BoT-Staple-CRH compared to the untargeted control (*p* < 0.005). No reduction in the release of ACTH was observed when AtT-20 cells were treated with the Bitox control (10 nM, data not shown).

### Selective targeting of neuronal populations

**Fig. 5:**
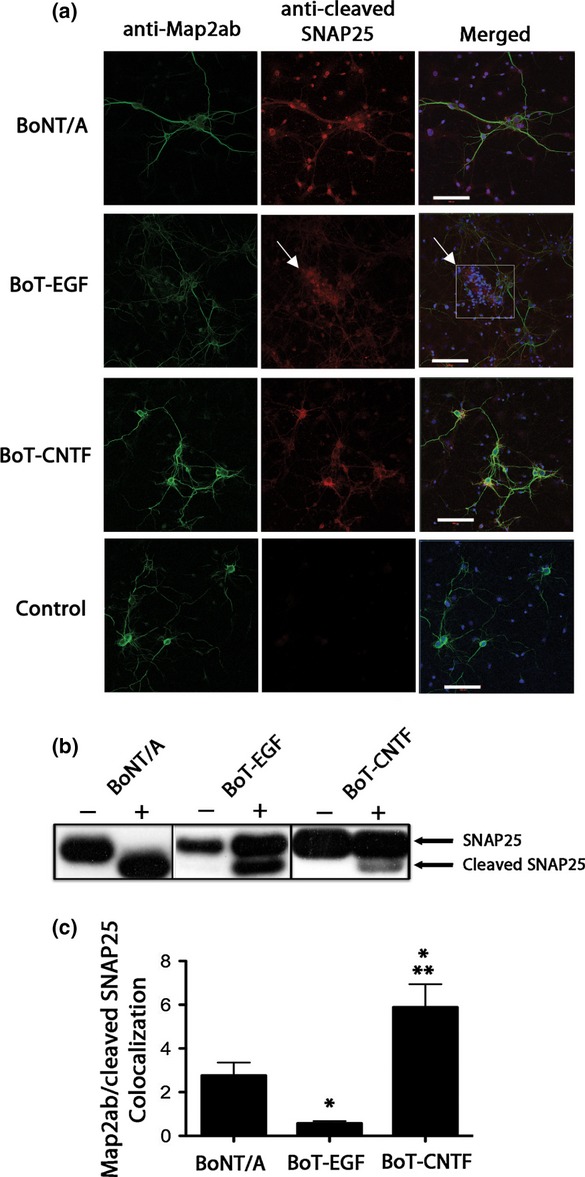
Differential targeting of neuronal populations by epidermal growth factor (EGF)- and ciliary neurotrophic factor (CNTF)-targeted botulinum molecules. (a) Confocal images of E18 rat cortical neurons treated with native BoNT/A, Botulinum enzymatic and translocation domains (BoT)-EGF and BoT-CNTF. The left column shows neurons immunostained with a Map2ab antibody (green), the middle column shows cleaved SNAP25 (red) and the right column shows the merge images, also containing nuclear Hoechst staining (blue). Horizontal bar: 100 μm (b) Immunoblot showing SNAP25 cleavage triggered by native botulinum neurotoxin (BoNT/A) and retargeted recombinant fusions. (c) Bar chart showing relative colocalization of the Map2ab and cleaved SNAP25 elicited by native and retargeted botulinum molecules (correlation coefficient multiplied by the signal intensity; ± SD). Significant differences can be seen between BoNT/A and BoT-EGF (*p < 0.005), BoNT/A and BoT-CNTF (**p < 0.03), as well as BoT-CNTF and BoT-EGF (*p < 0.005). BoT-CNTF preferentially delivers the botulinum protease into mature Map2ab-positive neurons whereas BoT-EGF acts preferentially on Map2ab-negative neuronal precursor cells.

Ligand-targeted BoTs may become useful not only in treatments of hypersecretory disorders but also for delineating and blocking specific neuronal subpopulations. We therefore investigated the ability of growth factor-directed BoTs to target rat cortical neurons in culture. BoT-affected neurons were visualized with the antibody against the cleaved SNAP25 (Fig. 5a). We used the dendritic marker Map2ab to distinguish mature neurons from neuronal precursors. The native BoNT/A cleaves the intracellular SNAP25 in both mature neurons (Map2ab+ cells) as well as neuronal precursors (Map2ab-/SNAP25+ cells) (Fig. 5a, top row). When assessed by western immunoblotting using the SMI81 anti-SNAP25 antibody, an almost total SNAP25 cleavage can be observed in the case of native BoNT/A, as for previously reported Bitox (Darios *et al*. 2010) (Fig. 5b). In contrast, only a proportion of SNAP25 is cleaved by BoT-EGF and BoT-CNTF (Fig. 5b). BoT-EGF (10 nM) predominantly targeted Map2ab negative cells, that is, immature neurons (Fig. 5a, second row) suggesting that neuronal stem cells together with transit amplifying cells and neuroblasts can be targeted by the BoT-EGF (Moyse *et al*. 2008). An abundant amount of cleaved SNAP25 signal can be evidenced in a proliferating ‘neurosphere’ (white arrow; Figure S3) where the neuronal precursor cells should carry abundant EGF receptors (Reynolds and Weiss 1992; Hermann *et al*. 2006; Moyse *et al*. 2008). Interestingly, when BoT-CNTF was applied to neocortical cultures, we mostly detected the cleaved SNAP25 signal in Map2ab-positive cells (Fig. 5a, third row). This agrees well with the expression in mature neurons of the CNTF receptor, which promotes differentiation, cell survival, and neurite outgrowth (Richardson 1994; Sleeman *et al*. 2000). The absence of cleaved SNAP25 in the control experiment (Fig. 5a, fourth row) confirmed the antibody specificity in cell immunostaining experiments. Fig. 5c shows quantification of co-localization of cleaved SNAP25 with Map2ab as observed by immunocytochemistry. BoT-CNTF has a high degree of colocalization with Map2ab, which significantly outweighs the native toxin (*p* < 0.03) and BoT-EGF (*p* < 0.005), suggesting targeting of predominantly mature neurons. BoT-EGF, on the other hand, has an inverse relationship with Map2ab+ cells and thus mainly targets precursor cells (*p* < 0.005).

## Discussion

Together, our results demonstrate that new ligands can substitute the botulinum receptor-binding domain and allow targeting of distinct neurons and cells of neuroendocrine origin. Recently, increasing efforts have been directed toward modifying several types of botulinum neurotoxins for treatment of diverse hypersecretory disorders including inflammation, asthma, chronic pain, and NETs, such as acromegaly and Cushing's disease (Chaddock *et al*. 2004; Foster 2005; Chaddock and Marks 2006; Foster *et al*. 2006; Chen and Barbieri 2009; Foster and Chaddock 2010; Pickett and Perrow 2011; Somm *et al*. 2012). Chimeric proteins have been designed with the aim of lowering systemic botulism toxicity and redirecting the botulinum activity toward the desired cells, for example the botulinum type C protease (Chaddock *et al*. 2000a,b; Foster *et al*. 2006). Currently the vast majority of botulinum-based therapies utilize specifically type A botulinum enzyme (e.g. BOTOX, Dysport, Xeomin preparations), which allows the longest known blockade of secretion, lasting up to 6 months. Here, we addressed the possibility of retargeting type A botulinum enzyme using a new approach – the protein stapling technique (Ferrari *et al*. 2012). When designing new chimeric proteins, direct fusion of protein modules can lead to misfolding, proteolytic degradation, and low yields. Recombinant fusion of chimeric proteins also has limitations because of the unidirectional nature of protein translation (N–C). Design of new biological molecules thus would benefit from a prior functional testing of separate units, which can be then combined on demand (Smith and Hecht 2011; Ferrari *et al*. 2012). Here, functional proteins carrying necessary linkers were produced separately, stapled together using a short synthetic peptide and then tested for their functionality (Darios *et al*. 2010). We now show that this approach allows successful delivery of the type A botulinum protease into specific neuroendocrine cells using both neuropeptides and growth factors.

Different cell types express a mosaic of cell-surface receptor including G-protein coupled receptors (GPCRs) and tyrosine kinase receptors that bind neuropeptides and growth factors. These receptors can internalize into the cell upon ligand binding and thus may be compatible for the delivery the botulinum molecules into the cytosol. Indeed, neuropeptides and growth factors have been used to deliver various cargoes into specific cells, but significant obstacles hamper utilization of these ligands (Fisher *et al*. 1996; Langer and Beck-Sickinger 2001; Middleton and Kellam 2005; Foster *et al*. 2006; Zhou *et al*. 2007; Frankel *et al*. 2009). It is well known that the binding activity of neuropeptides and growth factors often depends on post-translational modifications that could be difficult to implement. Another problem relates to poor expression of extracellular ligands in bacteria when designing new therapeutic chimeras based on bacterial proteins such as the botulinum protease.

Here, we investigated TNFα, CNTF, EGF, dermorphin, dynorphin 17, substance P, somatostatin, corticotropin-releasing hormone, and the native botulinum receptor-binding domain for their ability to direct the botulinum protease into a variety of neuroendocrine tumor cells. Each ligand was prepared with a linker sequence necessary for the stapling reaction. Upon ligand-dependent internalization into acidifying endosomes, the translocation domain enables the SNAP25 protease to reach and cleave its substrate (Harper *et al*. 2011). We found that EGF, CNTF, and CRH ligands were the most effective in delivering the botulinum protease into neuroendocrine cells. When we directly fused the wide range of ligands to the BoT unit we observed that only half of the ligands yielded workable recombinant fusions. EGF and CNTF direct fusions were as active on the neuroendocrine tumor cells as the stapled products in terms of selectivity and cleavage of SNAP25 (Figs. 2 and 3). The selectivity of our ligand-directed BoT molecules toward their cognate receptor systems was evidenced by reduction in SNAP25 cleavage in the presence of their native hormones. Overall, protein stapling allowed construction of a wider range of active botulinum products with varied structural diversity and better yields. Modifications such as C-terminal amidation of substance P, free N-terminus of the dermorphin peptide are important in targeting efficacy (Fisher *et al*. 1996). Using double targeting domains (2 × SP), on the other hand, can aid statistical rebinding of ligands and receptor dimerization can lead to a better penetration of the botulinum enzyme. In our attempts, we only observed an improvement in SNAP25 cleavage when using C-terminally amidated substance P or dermorphin when stapled to the botulinum protease. These observations are of interest because substance P and dermorphin have been used to target neuronal circuits involved in pain processing (Nichols *et al*. 1999; Ossipov *et al*. 2010). We envisage that botulinum-stapled substance P and dermorphin could provide long-term but reversible control of chronic pain states. Further exploration of secondary modifications and combinations of various ligands might yield improved targeting in future studies.

The BoT-EGF molecule developed in this study could be a prototype medicine for treatment of pheochromocytomas. These adrenal neoplasms are unresponsive to radio- or chemotherapy, and only treatable by surgery. Furthermore, during ablative operations, the neoplasms release unpredictable amounts of catecholamines, requiring constant monitoring and pharmacological fine-tuning to stabilize the patients (Cryer 1992; Garg and Banitt 2004; Lin *et al*. 2007; Low *et al*. 2012). Given that, the BoT-induced SNAP25 cleavage results in a secretory blockade, BoT-EGF could be beneficial not only for palliative care prior to surgery but also as a long-duration pharmacological intervention of non-malignant pheochromocytomas.

It has been estimated that up to 5% of the human population suffers from endocrine disorders (Golden *et al*. 2009) and long-term strategies to modulate hormonal secretion would be beneficial when invasive surgeries are unsuccessful or are associated with intolerable side effects. This study suggests that a wide range of long-acting botulinum therapies can become available when utilizing the protein stapling technology. Finally, we demonstrated in this study that the retargeted versions of botulinum protease can be used to dissociate separate populations of neurons. This opens a door for selective and tailored neuronal silencing in future research applications.

## Abbreviations

ACTH: adrenocorticotropic hormone
BoNT/A: botulinum neurotoxin serotype A
BoT: botulinum enzymatic and translocation domains
CNTF: ciliary neurotrophic factor
CRH: corticotropin releasing hormone
Dermo: dermorphine
DMEM: dulbecco's modified eagle medium
DMSO: dimethyl sulfoxide
Dyn17: dynorphin 17
EGF: epidermal growth factor
FCS: fetal calf serum
GST: glutathione S-transferase
HS: horse serum
NET: neuroendocrine tumors
NSF: *N*-ethylmaleimide sensitive fusion protein
P/S: penicillin and streptomycin
PAGE: polyacrylamide gel electrophoresis
PBS: phosphate buffer solution
SDS: sodium dodecyl sulfate
SNAP25: synaptosomal-associated protein 25
SNARE: soluble NSF-attachment protein receptors
SP: substance P
SS: somatostatin
TNFα: tumor necrosis factor α

## Supporting information

**Figure S1.** Immunoblot showing SNAP25 cleavage in E18 rat cortical neurons treated with the indicated stapled botulinum constructs as revealed using SMI81 antibody recognizing both intact and cleaved SNAP25.

**Figure S2.** Bar chart showing similar ^3^H-catecholamine uptake (counts per minute, CPM) by PC12 cells (± SD) treated or not with the indicated botulinum molecules.

**Figure 3.** A magnified region indicated by the white arrow in the panel 5a showing presence of cleaved SNAP25 in the ‘neurosphere’ following treatment with BoT-EGF.

## References

[b1] Amiche M, Delfour A, Nicolas P (1988). Structural requirements for dermorphin opioid receptor binding. Int. J. Pept. Protein Res.

[b2] Antonucci F, Rossi C, Gianfranceschi L, Rossetto O, Caleo M (2008). Long-distance retrograde effects of botulinum neurotoxin A. J. Neurosci.

[b3] Arsenault J, Cabana J, Fillion D, Leduc R, Guillemette G, Lavigne P, Escher E (2010). Temperature dependent photolabeling of the human angiotensin II type 1 receptor reveals insights into its conformational landscape and its activation mechanism. Biochem. Pharmacol.

[b4] Bajohrs M, Rickman C, Binz T, Davletov B (2004). A molecular basis underlying differences in the toxicity of botulinum serotypes A and E. EMBO Rep.

[b5] Bajohrs M, Darios F, Peak-Chew SY, Davletov B (2005). Promiscuous interaction of SNAP-25 with all plasma membrane syntaxins in a neuroendocrine cell. Biochem. J.

[b6] Bangaru MLY, Woodliff J, Raff H, Kansra S (2010). Growth Suppression of Mouse Pituitary Corticotroph Tumor AtT20 Cells by Curcumin: A Model for Treating Cushing's Disease. PLoS ONE.

[b7] Batcher E, Madaj P, Gianoukakis AG (2011). Pancreatic Neuroendocrine Tumors. Endocr. Res.

[b8] Binz T, Rummel A (2009). Cell entry strategy of clostridial neurotoxins. J. Neurochem.

[b9] Boyd RS, Duggan MJ, Shone CC, Foster KA (1995). The effect of botulinum neurotoxins on the release of insulin from the insulinoma cell lines HIT-15 and RINm5F. J. Biol. Chem.

[b10] Chaddock JA, Marks PM (2006). Clostridial neurotoxins: structure-function led design of new therapeutics. Cell. Mol. Life Sci.

[b11] Chaddock JA, Purkiss JR, Duggan MJ, Quinn CP, Shone CC, Foster KA (2000a). A conjugate composed of nerve growth factor coupled to a non-toxic derivative of Clostridium botulinum neurotoxin type A can inhibit neurotransmitter release in vitro. Growth Factors.

[b12] Chaddock JA, Purkiss JR, Friis LM, Broadbridge JD, Duggan MJ, Fooks SJ, Shone CC, Quinn CP, Foster KA (2000b). Inhibition of vesicular secretion in both neuronal and nonneuronal cells by a retargeted endopeptidase derivative of Clostridium botulinum neurotoxin type A. Infect. Immun.

[b13] Chaddock JA, Purkiss JR, Alexander FC (2004). Retargeted clostridial endopeptidases: inhibition of nociceptive neurotransmitter release in vitro, and antinociceptive activity in in vivo models of pain. Mov. Disord.

[b14] Chen S, Barbieri JT (2009). Engineering botulinum neurotoxin to extend therapeutic intervention. Proc. Natl Acad. Sci. USA.

[b15] Cryer PE (1992). Pheochromocytoma. West. J. Med.

[b16] Darios F, Niranjan D, Ferrari E (2010). SNARE tagging allows stepwise assembly of a multimodular medicinal toxin. Proc. Natl Acad. Sci. USA.

[b17] Davletov B, Bajohrs M, Binz T (2005). Beyond BOTOX: advantages and limitations of individual botulinum neurotoxins. Trends Neurosci.

[b18] Davletov B, Ferrari E, Ushkaryov Y (2012). Presynaptic neurotoxins: An expanding array of natural and modified molecules. Cell Calcium.

[b19] Ekong TA, Feavers IM, Sesardic D (1997). Recombinant SNAP-25 is an effective substrate for Clostridium botulinum type A toxin endopeptidase activity in vitro. Microbiology.

[b20] Fasshauer D, Sutton RB, Brunger AT, Jahn R (1998). Conserved structural features of the synaptic fusion complex: SNARE proteins reclassified as Q- and R-SNAREs. Proc. Natl Acad. Sci. USA.

[b21] Ferolla P, Faggiano A, Mansueto G (2008). The biological characterization of neuroendocrine tumors: the role of neuroendocrine markers. J. Endocrinol. Invest.

[b22] Ferrari E, Maywood ES, Restani L, Caleo M, Pirazzini M, Rossetto O, Hastings MH, Niranjan D, Schiavo G, Davletov B (2011). Re-Assembled Botulinum Neurotoxin Inhibits CNS Functions without Systemic Toxicity. Toxins (Basel).

[b23] Ferrari E, Soloviev M, Niranjan D, Arsenault J, Gu C, Vallis Y, O'Brien J, Davletov B (2012). Assembly of protein building blocks using a short synthetic Peptide. Bioconjug. Chem.

[b24] Fisher CE, Sutherland JA, Krause JE, Murphy JR, Leeman SE, vanderSpek JC (1996). Genetic construction and properties of a diphtheria toxin-related substance P fusion protein: in vitro destruction of cells bearing substance P receptors. Proc. Natl Acad. Sci. USA.

[b25] Foster KA (2005). A new wrinkle on pain relief: re-engineering clostridial neurotoxins for analgesics. Drug Discov. Today.

[b26] Foster K, Chaddock J (2010). Targeted secretion inhibitors-innovative protein therapeutics. Toxins (Basel).

[b27] Foster KA, Adams EJ, Durose L (2006). Re-engineering the target specificity of Clostridial neurotoxins - a route to novel therapeutics. Neurotox. Res.

[b28] Frankel AE, Woo J-H, Neville DM, Oldham RK, Dillman RO (2009). Immunotoxins. Principles of Cancer Biotherapy.

[b29] Gao Y, Zorman S, Gundersen G, Xi Z, Ma L, Sirinakis G, Rothman JE, Zhang Y (2012). Single reconstituted neuronal SNARE complexes zipper in three distinct stages. Science.

[b30] Garg A, Banitt PF (2004). Pheochromocytoma and myocardial infarction. South. Med. J.

[b31] Golden SH, Robinson KA, Saldanha I, Anton B, Ladenson PW (2009). Clinical review: Prevalence and incidence of endocrine and metabolic disorders in the United States: a comprehensive review. J. Clin. Endocrinol. Metab.

[b32] Harper CB, Martin S, Nguyen TH (2011). Dynamin inhibition blocks botulinum neurotoxin type A endocytosis in neurons and delays botulism. J. Biol. Chem.

[b33] Hermann A, Maisel M, Liebau S, Gerlach M, Kleger A, Schwarz J, Kim KS, Antoniadis G, Lerche H, Storch A (2006). Mesodermal cell types induce neurogenesis from adult human hippocampal progenitor cells. J. Neurochem.

[b34] Kirmani S (2012). Molecular genetic testing in endocrinology - a practical guide. Endocr. Pract.

[b35] Kroesen M, Lindau D, Hoogerbrugge P, Adema GJ (2012). Immunocombination therapy for high-risk neuroblastoma. Immunotherapy.

[b36] Langer M, Beck-Sickinger AG (2001). Peptides as carrier for tumor diagnosis and treatment. Curr. Med. Chem. Anticancer Agents.

[b37] Langley K (1994). The neuroendocrine concept today. Ann. N. Y. Acad. Sci.

[b38] Lin PC, Hsu JT, Chung CM, Chang ST (2007). Pheochromocytoma underlying hypertension, stroke, and dilated cardiomyopathy. Tex. Heart Inst. J.

[b39] Low G, Dhliwayo H, Lomas DJ (2012). Adrenal neoplasms. Clin. Radiol.

[b40] Mahrhold S, Rummel A, Bigalke H, Davletov B, Binz T (2006). The synaptic vesicle protein 2C mediates the uptake of botulinum neurotoxin A into phrenic nerves. FEBS Lett.

[b41] Martin TF, Grishanin RN (2003). PC12 cells as a model for studies of regulated secretion in neuronal and endocrine cells. Methods Cell Biol.

[b42] Meunier FA, Lisk G, Sesardic D, Dolly JO (2003). Dynamics of motor nerve terminal remodeling unveiled using SNARE-cleaving botulinum toxins: the extent and duration are dictated by the sites of SNAP-25 truncation. Mol. Cell. Neurosci.

[b43] Middleton RJ, Kellam B (2005). Fluorophore-tagged GPCR ligands. Curr. Opin. Chem. Biol.

[b44] Moyse E, Segura S, Liard O, Mahaut S, Mechawar N (2008). Microenvironmental determinants of adult neural stem cell proliferation and lineage commitment in the healthy and injured central nervous system. Curr. Stem Cell Res. Ther.

[b45] Nichols ML, Allen BJ, Rogers SD (1999). Transmission of chronic nociception by spinal neurons expressing the substance P receptor. Science.

[b46] Ossipov MH, Dussor GO, Porreca F (2010). Central modulation of pain. J. Clin. Invest.

[b47] Pickett A, Perrow K (2011). Towards new uses of botulinum toxin as a novel therapeutic tool. Toxins (Basel).

[b48] Prejbisz A, Lenders JW, Eisenhofer G, Januszewicz A (2011). Cardiovascular manifestations of phaeochromocytoma. J. Hypertens.

[b49] Reynolds BA, Weiss S (1992). Generation of neurons and astrocytes from isolated cells of the adult mammalian central nervous system. Science.

[b50] Richardson PM (1994). Ciliary neurotrophic factor: a review. Pharmacol. Ther.

[b51] Schiavo G, Santucci A, Dasgupta BR, Mehta PP, Jontes J, Benfenati F, Wilson MC, Montecucco C (1993). Botulinum neurotoxins serotypes A and E cleave SNAP-25 at distinct COOH-terminal peptide bonds. FEBS Lett.

[b52] Schiavo G, Matteoli M, Montecucco C (2000). Neurotoxins affecting neuroexocytosis. Physiol. Rev.

[b53] Shone CC, Melling J (1992). Inhibition of calcium-dependent release of noradrenaline from PC12 cells by botulinum type-A neurotoxin. Long-term effects of the neurotoxin on intact cells. Eur. J. Biochem.

[b54] Sleeman MW, Anderson KD, Lambert PD, Yancopoulos GD, Wiegand SJ (2000). The ciliary neurotrophic factor and its receptor, CNTFR alpha. Pharm. Acta Helv.

[b55] Smith BA, Hecht MH (2011). Novel proteins: from fold to function. Curr. Opin. Chem. Biol.

[b56] Somm E, Bonnet N, Martinez A (2012). A botulinum toxin-derived targeted secretion inhibitor downregulates the GH/IGF1 axis. J. Clin. Invest.

[b57] Südhof TC, Rothman JE (2009). Membrane Fusion: Grappling with SNARE and SM Proteins. Science.

[b58] Sutton RB, Fasshauer D, Jahn R, Brunger AT (1998). Crystal structure of a SNARE complex involved in synaptic exocytosis at 2.4 A resolution. Nature.

[b59] Taupenot L (2007). Analysis of regulated secretion using PC12 cells. Curr. Protoc. Cell Biol.

[b60] Thirunavukkarasusx N, Ghosal KJ, Kukreja R, Zhou Y, Dombkowski A, Cai S, Singh BR (2011). Microarray analysis of differentially regulated genes in human neuronal and epithelial cell lines upon exposure to type A botulinum neurotoxin. Biochem. Biophys. Res. Commun.

[b61] Tritos NA, Biller BM (2012). Advances in medical therapies for Cushing's syndrome. Discov. Med.

[b62] Yowler BC, Kensinger RD, Schengrund CL (2002). Botulinum neurotoxin A activity is dependent upon the presence of specific gangliosides in neuroblastoma cells expressing synaptotagmin I. J. Biol. Chem.

[b63] Zhou M, Nakatani E, Gronenberg LS, Tokimoto T, Wirth MJ, Hruby VJ, Roberts A, Lynch RM, Ghosh I (2007). Peptide-labeled quantum dots for imaging GPCRs in whole cells and as single molecules. Bioconjug. Chem.

