# Supplementary material for: Stapling of the botulinum type A protease to growth factors and neuropeptides allows selective targeting of neuroendocrine cells
Source: J Neurochem. 2013 May 20;126(2):223–33. doi: 10.1111/jnc.12284 (PMC3758956; doi:10.1111/jnc.12284)

Arsenault *et al*, 'Stapling of the botulinum type A protease to growth factors and neuropeptides allows selective targeting of neuroendocrine cells'

SUPPORTING INFORMATION:

Supplementary figure 1: Immunoblot showing SNAP25 cleavage in E18 rat cortical neurons treated with the indicated stapled botulinum constructs as revealed using SMI81 antibody recognizing both intact and cleaved SNAP25.

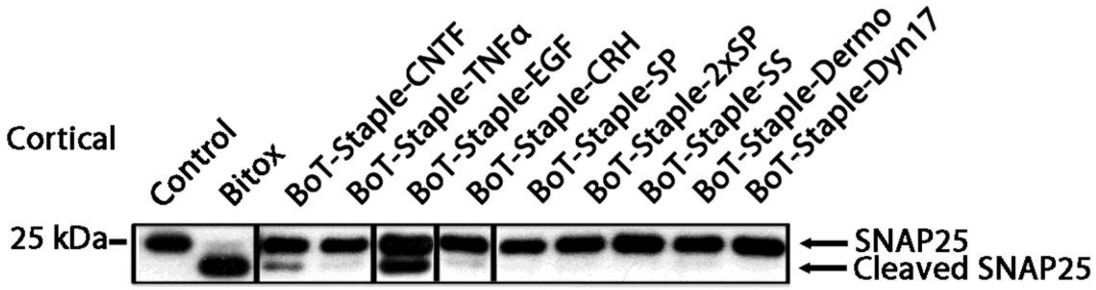

Supplementary figure 2: Bar chart showing similar ^{3}H-catecholamine uptake (counts per minute, CPM) by PC12 cells (+/- SD) treated or not with the indicated botulinum molecules.

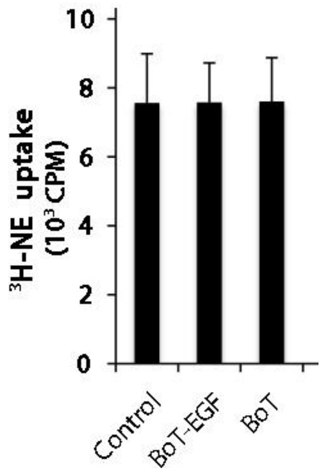

Arsenault *et al*, 'Stapling of the botulinum type A protease to growth factors and neuropeptides allows selective targeting of neuroendocrine cells'

Supplementary figure 3: A magnified region indicated by the white arrow in the panel 5a showing presence of cleaved SNAP25 in the 'neurosphere' following treatment with BoT-EGF. Neurons were immunostained with an anti-Map2ab (green) and the anti-cleaved SNAP25 (red) antibodies. Nuclear Hoechst staining is shown in blue. Horizontal bar: 50 μm.

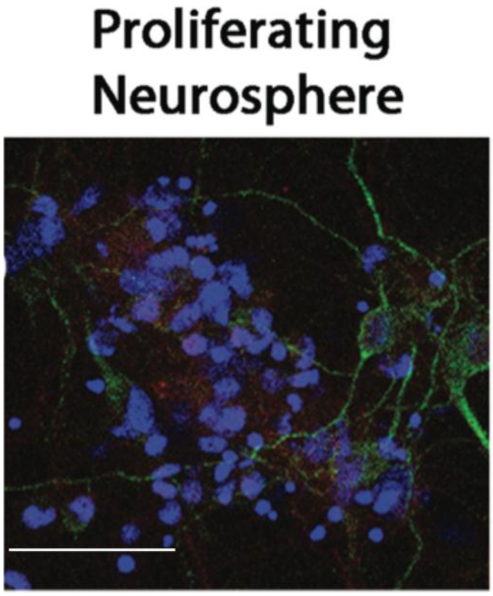

Supplement: Supplementary file 1 [file jnc0126-0223-SD1.pdf]
